# Capillary malformations

**DOI:** 10.1172/JCI172842

**Published:** 2024-04-15

**Authors:** Adrienne M. Hammill, Elisa Boscolo

**Affiliations:** 1 Division of Hematology, Cincinnati Children’s Hospital Medical Center, Cincinnati, Ohio, USA.; 2 Department of Pediatrics, University of Cincinnati College of Medicine, Cincinnati, Ohio, USA.; 3 Division of Experimental Hematology and Cancer Biology, Cincinnati Children’s Hospital Medical Center, Cincinnati, Ohio, USA.

## Abstract

Capillary malformation (CM), or port wine birthmark, is a cutaneous congenital vascular anomaly that occurs in 0.1%–2% of newborns. Patients with a CM localized on the forehead have an increased risk of developing a neurocutaneous disorder called encephalotrigeminal angiomatosis or Sturge-Weber syndrome (SWS), with complications including seizure, developmental delay, glaucoma, and vision loss. In 2013, a groundbreaking study revealed causative activating somatic mutations in the gene (*GNAQ*) encoding guanine nucleotide–binding protein Q subunit α (Gαq) in CM and SWS patient tissues. In this Review, we discuss the disease phenotype, the causative *GNAQ* mutations, and their cellular origin. We also present the endothelial Gαq-related signaling pathways, the current animal models to study CM and its complications, and future options for therapeutic treatment. Further work remains to fully elucidate the cellular and molecular mechanisms underlying the formation and maintenance of the abnormal vessels.

## Introduction

**Figure 1:**
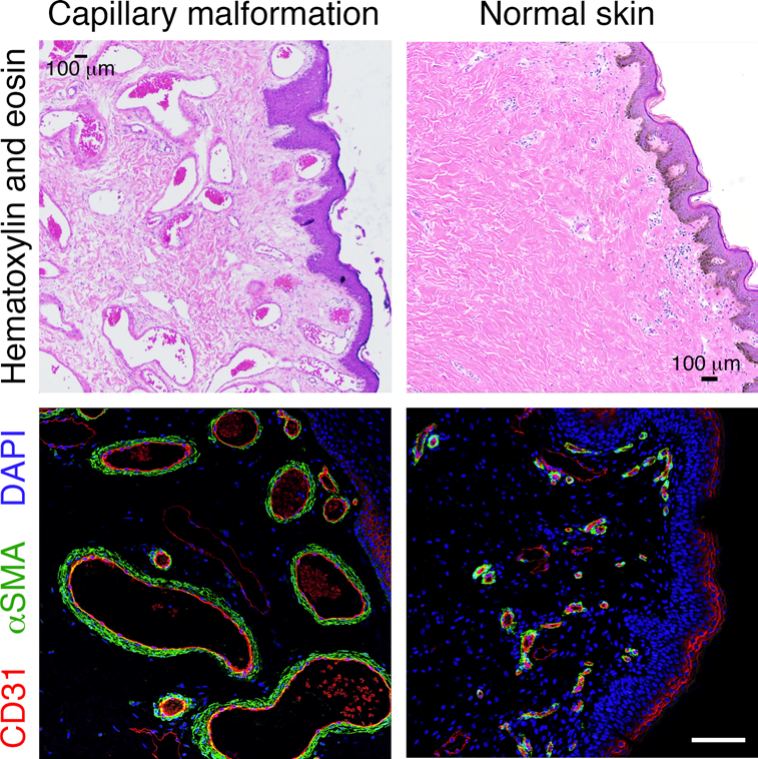
Histopathology of capillary malformation tissue. Tissue from a CM/SWS patient (left) and control normal skin (right), stained with H&E (top) and for CD31 (red)/αSMA (green)/DAPI to label nuclei (blue) (bottom). Scale bars: 100 μm.

Capillary malformations (CMs), or port wine birthmarks, are congenital slow-flow vascular malformations characterized by increased numbers of dilated capillary blood vessels (Figure 1). CM occurs in 0.1%–2% of all newborns, with no sex differences (1).

CMs can occur on skin and mucosal surfaces anywhere in the body and are most noted on the face. CMs localized on the skin appear as light pink–to-red patches during childhood, with changes over time including darkening, thickening, and development of nodularity. CM can result in functional concerns such as lip overgrowth, resulting in difficulties eating or dental complications; or with thickening of the eyelid, making it difficult to close completely. CM has negative impact on health-related quality of life (2) and represents a source of stigma for many patients (3), resulting in psychological morbidity (4) and distress for both patients and families (5).

While isolated CM skin lesions are not life-threatening, they may indicate underlying pathology, depending on their location. The appearance of CMs on the face, particularly on the upper face and forehead area (6), can be associated with Sturge-Weber syndrome (SWS). CM lesions that occur over the midline back can be associated with spinal anomalies (7, 8). Furthermore, CM in combination with other vascular anomalies and overgrowth or undergrowth can be part of other syndromes, including Klippel-Trenaunay syndrome and other PIK3CA-related overgrowth spectrum (PROS) disorders. For purposes of this Review, we will focus on “cutaneous and/or mucosal CMs,” as described in the most recent classification from the International Society for the Study of Vascular Anomalies (9).

SWS is a known association between facial CM and involvement of the eye, brain, or both on the ipsilateral side(s). Eye involvement most commonly manifests as glaucoma, or increased intraocular pressure, but can also present with choroidal hemangioma, a proliferation of capillaries within the eye that can lead to retinal detachment and loss of vision. Untreated glaucoma can lead to optic nerve damage and loss of visual acuity. Sometimes increased intraocular pressure develops prenatally and buphthalmos, an enlarged eyeball, can be present at birth. Brain involvement in individuals with SWS appears as capillary venous malformation most commonly of the leptomeninges. This is classically visualized as leptomeningeal enhancement on contrast MRI and can present with a range of severity, from focal areas to involvement of full hemispheres. Cortical atrophy and intracranial calcifications may also be present, although not always visible on initial imaging (10, 11). Additional vascular abnormalities, including choroid plexus enlargement, with or without venous anomalies, and/or extensive deep draining vessels, are also suggestive of SWS. Brain involvement in SWS can manifest as seizure, headache, stroke or stroke-like episodes, developmental delays, cognitive limitations, and attention difficulties.

## Histopathology of CM

CMs are composed of immature venule-like channels that are negative for glucose transporter 1 (GLUT-1), which is an immunodiagnostic marker for infantile hemangioma (12, 13). The malformed blood vessels exist primarily in the papillary layer and upper reticular dermis, rarely extending into the deep dermis or subcutaneous fat layer. The histopathology of CM lesions is characterized by dilated vessels with irregular shapes (Figure 1). In a study from Tan et al., analysis of vessel size in CM lesions in comparison to normal skin showed a decreased number of small vessels (circumference 0–150 μm), which was associated with an increased number of large vessels (circumference 500–1000 μm) (14). This study also revealed that CM vessels are characterized by multiple layers of pericytes, thickened basement membrane, and disorganized collagen and elastic fibers, resulting in an overall thickened blood vessel wall. These pathological changes in the vessel walls are believed to occur prior to the dilation of the CM blood vessels. Sections of adult CM patient tissue have shown thin-walled blood vessels surrounded by flat, elongated endothelium (14). These findings would suggest that thick-walled rather than thin-walled blood vessels could be the primary pathological phenotypes during the early development of CM.

Tan and colleagues suggested that CM originates from immature endothelial cells (ECs), as CM blood vessels express stem cell markers such as CD133 and CD166. Additionally, ECs in CM show expression of both venous and arterial markers EphrinB1 and EphrinB2, respectively (14). It is interesting to speculate that the causative mutation happens during development in the primary capillary plexus and impairs definitive differentiation into dermal arterioles and venules.

## Genetic mutations in CM

In CM, as in other vascular malformations and tumors, vascular lesions usually have an asymmetric pattern or involve a defined body region. Rudolf Happle hypothesized that these types of sporadic lesions are caused by somatic mosaic mutations (15). Mosaicism in an individual denotes the presence of 2 or more clones of cells with distinct genetic variants, all of which are derived from a single fertilized egg. Mosaicism can be the result of an early mutational event happening during embryonic development or after birth. As a result of this process, cells expressing the postzygotic variant can then undergo clonal selection and proliferation to generate the vascular lesion (16). In 2009, a study led by Miikka Vikkula reported the discovery of somatic mutations in the *TEK* gene in venous malformation tissue from patients (17). This finding propelled the field to multiple subsequent discoveries of somatic mosaic mutations in most types of vascular anomalies (reviewed in ref. 18).

In the case of CM, a groundbreaking study in 2013 by Shirley and colleagues discovered a hyperactive, somatic nonsynonymous single-nucleotide variant (c.548G>A; p.R183Q) in the gene (*GNAQ*) encoding guanine nucleotide–binding protein Q subunit α (Gαq) in affected tissue from 88% of syndromic (SWS) and 92% of nonsyndromic CMs. The prevalence of the mutant allele, also called variant allele frequency (VAF), in affected tissues ranged from 1.0% to 18.1% (19). Couto, Huang, et al. further confirmed the presence of the *GNAQ* p.R183Q mutation in 10 of 13 CM/SWS patient tissues. Additionally, they reported two novel substitutions in the same locus: c.548G>T; p.R183L and c.547C>G; p.R183G (20). To date, multiple studies have confirmed the presence of mutations at the *GNAQ* p.R183 locus in approximately 71%–80% of the CM patients with VAF between 1.2% and 33.3% (21–23).

Mutations in *GNA11*, a highly related member of the Gαq protein family, have also been identified in CM patients at the same conserved site, p.Q183, although they are preferentially associated with reticulated CM in extremities, which present with hypotrophy or hypertrophy (24–26).

Recently, a somatic missense mutation in *GNB2* (c.232A*>*G; p.K78E) was identified in 1 patient with CM/SWS. *GNB2* encodes the β subunit of the heterotrimeric G protein and the p.K78E mutation promotes constitutive activation of the Gαq signaling pathway (27).

Finally, additional mutations have been identified in a few CM/SWS cases, such as *GNAQ* p.Q209R. The p.Q209R mutation has been shown to be a stronger activator compared with p.R183Q and a milder activator compared with the p.Q209L mutation that is associated with vascular tumors (28).

## *GNAQ* mutations in other vascular anomalies

Somatic hyperactive *GNAQ* mutations have also been identified in other types of vascular anomalies, including benign and locally aggressive vascular tumors. *GNAQ/11* mutations have been reported in approximately 75% of congenital hemangioma (CH), including both noninvoluting CH and rapidly involuting CH. In these patients, the most frequent substitution is at the p.Q209 site (29, 30). Other vascular tumors associated with hyperactive *GNAQ/11/14* mutations include tufted angioma, kaposiform hemangiothelioma, pyogenic granuloma, and anastomosing, papillary, and cherry hemangioma subtypes (31–36). While *GNAQ/11/14* mutations were identified with high frequency in patients with benign neoplasms, these gene mutations have not been reported in aggressive vascular tumors such as angiosarcoma or Kaposi’s sarcoma, suggesting hyperactive Gαq signaling in the endothelium does not participate in oncogenic transformation (33).

## *GNAQ* gene function

The *GNAQ* gene (37) encodes Gαq, a member of the heterotrimeric G-protein superfamily that exert a crucial function as proximal effectors of G protein–coupled receptor (GPCR) signaling, which is initiated by ligand binding.

There are four classes of Gα protein (Gαs, Gαq, Gαi, Gα12/13) and each class interacts with different downstream effectors. GPCRs can signal through more than one type of Gα protein (38). Vascular GPCRs signaling through Gαq include endothelin receptors (39), protease-activated-receptors (40), bradykinin receptors (40), and angiotensin-II type I receptor (41).

The Gαq protein family includes ubiquitously expressed Gαq and Gα11, and their closely related members Gα14 and Gα15, which show tissue-specific distribution. Knockout of any combination of both *Gnaq* and *Gna11* can be lethal in mice (42–44), suggesting that Gαq and Gα11 have redundant roles. Studies on EC-restricted deficiency of Gαq or Gαq/11 showed reduced EC proliferation and retinal angiogenesis (45), establishing the important role of Gαq signaling during vascular development.

The Gαq protein is composed of two structural domains: a highly conserved nucleotide-binding domain (Ras-like GTPase domain) and a helical domain. Nucleotide-dependent conformational changes occur primarily within three flexible regions called switch I, switch II, and switch III (46). The *GNAQ/11* mutation at p.R183 is localized in the switch I region. In the resting state, the Gαq subunit is anchored to the cell membrane and forms a heterotrimeric complex with the β and γ subunits. In response to GPCR activation, the α subunit serves as a molecular switch for the G protein, which is active when bound to GTP and is inactivated when GTP is hydrolyzed to GDP (47, 48). The enzymatic activity of the α subunit promotes its dissociation from the heterotrimeric complex (49). Both GTP-bound Gα and free Gβγ are capable of initiating signals by interacting with downstream effector proteins. The unbound Gα subunit can mediate signaling until its GTP is hydrolyzed back to GDP and it reassociates with the β and γ subunits. This inactivation step is modulated by regulators of G-protein signaling proteins, which accelerate the rate of GTP hydrolysis and thereby limit the half-life of the activated signals (50). β-Arrestins can also bind to GPCRs to inhibit binding to heterotrimeric G-proteins and promote GPCR endocytosis (51).

Gain-of-function hyperactive mutant forms of Gαq and Gα11 are known oncogenic drivers in uveal melanoma, an aggressive cancer of the eye (52). In uveal melanoma, approximately 95% of the *GNAQ* or *GNA11* mutations affect the p.Q209 site, while 5% affect p.R183. Although *GNAQ* p.R183Q is an oncogenic mutation, in CM there is no evidence of accumulating mutations or metastatic events. While both *GNAQ* p.R183Q and p.Q209L are gain-of-function constitutively active mutants, they are located in the switch I and switch II protein domains, respectively, and computational studies revealed that these mutations may confer a differential impact on the Gαq protein structure (53). The p.R183Q mutation was predicted to abolish hydrogen bonds between the R183 residue and GDP molecule, destabilizing the inactive GDP-bound conformation of Gαq. The p.Q209L mutation was instead predicted to affect the molecular interaction between Gαq and Gβ subunit, impairing formation of the inactive heterotrimeric complex. These findings, in association with protein-protein interaction network analysis, indicate that p.R183Q and p.Q209L mutations may result in the overactivation of different downstream effectors, which in turn will determine the distinct cell responses and phenotype.

Similarly, the *GNB2* mutation identified in one CM patient is predicted to disrupt a salt bridge bond between the Gα and the Gβγ subunits, impairing GTP hydrolysis and promoting a constitutive Gαq signaling pathway (27).

Activating somatic *GNAQ* mutations have also been identified in melanocytic blue nevi and nevi of Ota (52). Postzygotic activating mutations in *GNA11* or *GNAQ* can also cause phakomatosis pigmentovascularis (54), a group of conditions defined by the presence of both pigmentary and vascular birthmarks (55, 56). In mice, activating *GNAQ/11* germline mutations (*GNAQ* p.V179M and *GNA11* p.I63V) can cause dermal hyperpigmentation (57). The abnormal early melanocytic development resulting from these mutations in neural crest cells is mediated through endothelin. Endothelin has important roles in vasculogenesis and vascular tone, implying that dysregulation of this GPCR signaling pathway could be a contributor to vascular malformations with Gαq/11 mutations. In addition, SWS, CM, and melanocytic nevi are likely to originate during development; therefore, the effects of the same *GNAQ* somatic mutation may be different depending on the cell type and timing of the mutational event during development.

## Cellular origin of *GNAQ* mutation

*GNAQ* is expressed in different cell types, including ECs, neurons, alveolar cells in the lung, and blood mononuclear cells.

In CM patients, *GNAQ/11* mutations are somatic (i.e., noninherited) and the allelic frequency in the patients’ affected tissue is generally quite low, suggesting the mutational event affects only one cell or a small cell population. The origin of the *GNAQ* mutation in CM lesions was first identified in a study from Couto et al. (20). In this study, lesional cells from CM patients were analyzed by FACS and four cell populations were sequenced: hematopoietic cells (CD45^+^); ECs expressing VE-cadherin, CD31, VEGFR2, and/or CD34; pericytes/smooth muscle cells (PDGFRβ^+^); and stromal cells (endothelial cell marker^–^, PDGFRβ^–^). The ECs showed the highest VAF, ranging from 2.8% to 42.9%. A follow-up study with lesional brain samples also demonstrated significant enrichment of mutational allelic frequencies in ECs compared with the other cell populations (58). Another study by Couto and colleagues established a positive correlation between the mutant allele frequency in the EC population and the disease severity (20). This could have important implications for predicting the natural history of a lesion based on analysis of CM tissue biopsies. It is worth noting that in a small subset of the patients analyzed for both skin and brain lesions, the heterogeneous stromal cell population (which may contain fibroblasts and undifferentiated cells) also contained mutant cells. This could be due to (a) possible presence of uncaptured ECs and/or (b) existence of mutant pluripotent cells. The second hypothesis is substantiated by other studies reporting the *GNAQ* mutation in connective tissues, hair follicles, and glands in CM (59).

## Models of CM/SWS and other *GNAQ*-related vascular anomalies

Animal models are essential tools for understanding disease processes and for testing novel therapeutic targets. Since the identification of the CM causative mutation, few animal models have been reported to date. The first animal model of CM, reported by Huang and colleagues, is a xenograft (60). Xenograft models consist of the injection or transplantation of patient-derived cells or tissues into mice or utilize patient-specific platforms such as human cells expressing the genetic mutation identified in patients. Xenografts have proven to be very valuable tools in the cancer and vascular anomalies fields to test drug treatments for precision medicine (61, 62).

In generating their model of CM, Huang and colleagues utilized endothelial colony-forming cells (ECFCs) engineered to express *GNAQ* p.R183Q (60). ECs were mixed with bone marrow mesenchymal progenitor cells to serve as blood vessel–supporting cells and mixed with a solubilized basement membrane matrix prior to subcutaneous injection into immunocompromised nude mice. Compared with ECs expressing WT *GNAQ*, mutant ECs formed blood vessels of increased circumference. Vessel size distribution analysis showed that in ECFC-R183Q versus ECFC-WT, the percentage of small-sized vessels (<100 μm circumference) is reduced, while the percentage of larger-sized vessels (101–300 μm circumference) is increased, mirroring CM patient vessels compared with normal skin (14, 60). This xenograft model was the first to show that endothelial *GNAQ* p.R183Q is sufficient to drive formation of CM-like vessels.

A second xenograft model by Sasaki and colleagues, based on murine ECs expressing *GNAQ* p.Q209L, confirmed that the *GNAQ*-mutant ECs could form enlarged, ectatic vessels upon subcutaneous injection into mice (63).

While xenografts have proven very useful for modeling the mosaic nature of the vascular lesions, additional strategies to model the congenital nature of CM formation should focus on genetic models that can recapitulate early events happening during embryonic development. Recently, Wetzel-Strong and colleagues (64) developed a mouse system allowing for conditional expression of *Gnaq* p.R183Q from the endogenous *Gnaq* locus. In this model, expression of the mutant allele is regulated by Cre recombination. *Gnaq* p.R183Q mice were crossed with a mosaic global *E2a-*Cre or ubiquitous global *β-actin*-Cre mouse line. Analysis of mutant embryos at E13.5–14.5 showed disease-relevant vascular defects such as edema, dilated vascular channels, and hemorrhage. Of interest, while ubiquitous global expression of *Gnaq* p.R183Q with the *β-actin*-Cre mouse line resulted in complete embryonic lethality, use of the mosaic *E2a*-Cre mouse line produced partial embryonic lethality. These findings strongly support Happle’s hypothesis that germline inheritance of the *GNAQ* p.R183Q mutation would be lethal, while somatic mosaic expression can allow for survival.

Similar findings by Schrenk and colleagues (65) showed that global ubiquitous expression (driven by *CMV*-Cre) of the *GNAQ* p.Q209L allele resulted in embryonic lethality. In this model, lethality occurred before E8.5, compared with *Gnaq* p.R183Q in which lethality occurred between E14.5 and P0. This suggests that ubiquitous expression of the p.Q209L mutation, which is a stronger activating mutation, leads to a more severe phenotype leading to lethality earlier than p.R183Q. However, it is important to keep in mind that different ubiquitous Cre-driver mouse lines were used in the two studies and the genetic murine model based on p.Q209L is a transgenic model with possible overexpression of the mutant allele by the endogenous Rosa26 promoter. In the Schrenk et al. study (65), the *GNAQ^Q209L^* allele was also induced specifically in ECs by crossing *Rosa26-floxed stop-GNAQ^Q209L^* mice (66) with *Cdh5-iCreER^T2^* (67) or *Pdgfb-iCreER^T2^* (68) mice. Tamoxifen-mediated activation of the mutant *GNAQ* in pups or adult mice resulted in aberrant vascular morphogenesis in the subcutaneous tissue, brain, and retina. Blood vessels of capillary-venous origin were dilated, similar to those seen in CM, and additionally formed vascular tufts, a common histopathological feature of vascular tumors with the *GNAQ* p.Q209L mutation.

Another model of Gαq hyperactivation in ECs is the Gαq-DREADD mice (69) (DREADDs are designer receptors exclusively activated by designer drugs; ref. 70), which express a modified M3 muscarinic receptor (hM3Dq) crossed with *Pdgfb-iCreER^T2^*. In this model, Cre^+^ mutant pups treated with clozapine *N*-oxide (specific activator of Gαq-DREADD) showed increased vascularity and vessel dilation in the skin, a phenotype resembling CM (65).

To better understand the cellular and molecular mechanisms underlying CM formation, future efforts should focus on genetic models with mosaic endothelial expression of *GNAQ* p.R183Q during embryonic development. It will be important to test the consequences of mutant *GNAQ* expression starting at different developmental time points and in different tissues. Furthermore, it is possible that targeting mutant allele expression to ECs may not be sufficient to recapitulate CM pathogenesis, suggesting that a progenitor or a stromal cell type could contribute to the origin of CM.

## Mutant-*GNAQ* signaling pathways

### Canonical and noncanonical signaling.

**Figure 2:**
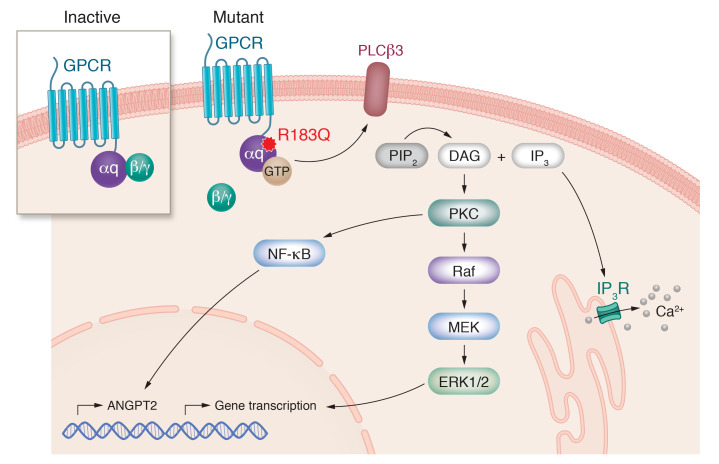
Mutant *GNAQ* p.R183Q signaling in ECs. Schematic of the molecular pathways involved in CM. The *GNAQ* activating mutation p.R183Q promotes disassembly of the heterotrimeric complex subunit αq from the β and γ subunits, impairing the hydrolysis of GTP to GDP. The Gαq bound to GTP promotes phospholipase C-β3 (PLCβ3) signaling, which catalyzes the conversion of phosphatidylinositol 4,5-bisphosphate (PIP_2_) to inositol trisphosphate (IP_3_) and diacylglycerol (DAG). DAG leads to the activation of protein kinase C (PKC), which can in turn induce RAF (rapidly accelerated fibrosarcoma) kinase activation and MEK, which phosphorylates ERK1 and -2, promoting translocation into the nucleus and gene transcription. PKC can also induce NF-κB translocation into the nucleus, inducing ANGPT2 expression to promote proangiogenic signaling. The IP_3_ metabolite can activate its receptor, IP_3_R, on the endoplasmic reticulum, stimulating the release of calcium into the cytoplasm.

GTP-bound Gαq can exert canonical signaling through phospholipase C-β (PLCβ) (71). Phosphorylation of PLCβ catalyzes the conversion of phosphatidylinositol 4,5-bisphosphate (PIP_2_) to inositol trisphosphate (IP_3_) and diacylglycerol (DAG). IP_3_ can then activate its receptor on the endoplasmic reticulum, stimulating the release of calcium into the cytoplasm. The concomitant production of DAG with the release of calcium leads to the activation of PKC. PKC can in turn activate RAS, a GTPase that can promote recruitment of RAF kinase on the membrane. Activated RAF acts on MEK, which phosphorylates the kinases ERK1 and -2 (Figure 2). Additional MEK effectors are c-Jun, JNK, and p38 kinase (72).

In ECs expressing *GNAQ* p.R183Q, PLCβ3 is constitutively activated by phosphorylation at Ser537 (60). Although increased MEK and ERK1/2 phosphorylation was documented in SWS patient tissue biopsies (73), the activation of the MAPK/ERK pathway in cultured ECs with the p.R183Q mutation is mild compared with ECs expressing *GNAQ* p.Q209L (19, 36, 54). Furthermore, the *GNAQ* p.R183Q substitution does not activate p38 or JNK in the same way that p.Q209L does (19, 54). The moderate activation of ERK, and differential effect on p38 and JNK pathways in ECs during fetal development, may explain the CM phenotype, as opposed to a vascular tumor.

Parallel to the canonical PLCβ-mediated signaling axis, Gαq has been shown to control noncanonical signaling through members of the Trio family of guanine nucleotide exchange factors for Rho, such as Trio, Kalirin, p63RhoGEF (74–76), and G protein receptor kinase 2 (GRK2), which bind to activated Gαq with high affinity (77, 78). In melanocytes expressing *GNAQ* p.Q209L, the activation of Trio leads to the phosphorylation of FAK, promoting the aberrant activation of YAP and PI3K pathways to drive tumor growth (79–83). To date, the activation of the noncanonical pathways, including YAP and PI3K, has not been established in ECs with hyperactive mutant *GNAQ*.

### Calcium signaling.

The role of mutant Gαq protein family members in calcium signaling is highlighted by the presence of inherited germline *GNA11* mutations in patients with hypoparathyroidism and hypocalciuric hypercalcemia. Different types of amino acid substitution in *GNA11* have been shown to contribute to the altered sensitivity of cells to extracellular calcium levels (84). In particular, loss-of-function *GNA11* mutations lead to elevated serum calcium concentrations, while gain-of-function mutations result in the opposite calcium signaling phenotype, which consists of low levels of calcium in the serum. Mice with biallelic germline deletion of *Gna11* and parathyroid-specific loss of *Gnaq* alleles also developed hypercalcemia, strongly implicating *GNAQ/11* signaling in extracellular calcium homeostasis in both humans and mice (85). Molecular analysis of the mutation *GNA11* p.R60L, which was identified in a patient with hypoparathyroidism, revealed it is less activating than *GNA11* p.Q209L (86). This supports the hypothesis that *GNA11* p.R60L leads to a less severe phenotype that is compatible with life when expressed in the germline compared with substitutions at p.Q209 and most likely at p.R183.

It is important to note that approximately 50% of patients with hypocalcemia type 1 are affected by seizures, which is a phenotypic manifestation of SWS (84). In most of these patients, seizures were associated with ectopic and basal ganglia calcifications. Furthermore, serum hypocalcemia has been associated with seizures, as low calcium concentrations in the cerebrospinal fluid can lead to increased excitability in the central nervous system (87).

Brain cortical calcifications have been reported in the majority of patients with SWS, as well as in other vascular anomalies such as cerebral cavernous malformation (CCM) and in genetic disorders affecting the RAS/MAPK pathway such as neurofibromatosis (88, 89).

In past years, conflicting reports made it unclear whether the calcifications in SWS patients are localized in the walls of the angiomatous vessels or are free parenchymal deposits within the superficial or deep cortex (90, 91). A recent study by Knöpfel and colleagues revealed that SWS patients have an abnormal calcium metabolic profile resulting in hypocalcemia (92), which correlated with neurological symptoms. Histopathological analysis of tissue biopsies determined that the calcium deposits were mostly in the vessel wall of cortical capillaries and small venules.

Furthermore, Zecchin and colleagues determined that expression of the *GNAQ* or *GNA11* p.R183Q mutation in human ECs increased the GPCR-induced intracellular calcium signaling, which is potentiated by the calcium release–activated calcium (CRAC) channels (93). Treatment with a short interfering RNA (siRNA) targeting the mutant allele or with a CRAC channel inhibitor promoted rescue of the increased calcium signaling.

Combined, these findings suggest that the progressive mineral deposition in the microvasculature contributes to the abnormal cerebral perfusion and neurological symptoms of SWS.

### Angiogenesis pathway signaling.

G-protein noncanonical functions include RTK signaling. VEGF-A signaling is a well-known regulator of EC mitogenic responses and promotes vascular migration and permeability by binding to its receptor KDR (kinase insert domain receptor). KDR was shown to be overexpressed in CM vessels, suggesting increased pathway activation that could contribute to the proangiogenic phenotype (94). Gαq/11 proteins can play a role in VEGF-induced EC migration by promoting KDR-mediated RhoA and Rac1 activation. Upon VEGF stimulation, KDR can form a rapid but transient complex with Gaq/11 (95). Next, upon Gαq/11 activation, the release of free Gβγ subunits can phosphorylate PLC and subsequently PKC, which in turn, leads to RhoA activation (96). Interaction between Gαq/11 proteins and KDR has also been shown to promote EC proliferation by mediating concomitant MAPK activation and intracellular calcium mobilization. While KDR activation by VEGF-A can promote Gαq/11 signaling, the stimulation of the bradykinin B2 receptor, a GPCR coupled with Gαq/11, induces tyrosine phosphorylation of KDR, and can promote increased endothelial nitric oxide (NO) synthase activity, which is a known regulator of vasodilation (97). This body of literature documents the important role of Gαq signaling during vascular development and supports the notion that excessive or reduced Gαq signaling leads to vascular abnormalities.

Angiogenesis and vascular permeability are also mediated by angiopoietin-2 (ANGPT2) (98, 99). ANGPT2 is expressed at low levels in quiescent ECs and is increased during angiogenesis and in response to inflammatory mediators, which leads to permeable and destabilized blood vessels. Although the precise molecular regulation of ANGPT2 by Gαq/11 is not known, several studies showed the overexpression of ANGPT2 in ECs expressing hyperactive mutant *GNAQ* (28, 60, 65). Knockdown of ANGPT2 in *GNAQ*-mutant (R183Q) ECs reduced blood vessel diameter in a xenograft model of CM, indicating an important role for ANGPT2 in the dilated vascular phenotype. Importantly, ANGPT2 has also been implicated in several other vascular anomalies such as arteriovenous malformations (AVMs) (100), CCM (101), and kaposiform lymphangiomatosis (KLA) (102, 103). Combined, these studies establish ANGPT2 as a promising potential common target for these vascular diseases.

ANGPT2 is also overexpressed during inflammation (104). Inflammatory markers such as NF-κB, IL-1β, and E-selectin were reported to be increased in ECs expressing *GNAQ* p.R183Q or p.Q209L (28, 60, 65). This suggests that EC-autonomous inflammatory processes are a common feature of ECs expressing hyperactive *GNAQ* and may in turn regulate ANGPT2 expression. Although different *GNAQ* mutations such as p.R183Q and p.Q209L are associated with different classes of vascular anomalies (as CM and vascular tumors, respectively), recent studies highlighted that the transcriptional consequences of these mutations in ECs are similar and include upregulation of pathways such as MAPK, angiogenesis, inflammation, and upregulation of *ANGPT2* (28, 60, 65). This would suggest that the differences between hyperactive *GNAQ* mutation types may affect the level of activation/expression rather than the specific downstream targets (28).

Finally, while the precise mediators of NF-κB activation in the context of CM are not known, one interesting possibility is that it can be activated by shear stress upon Gαq activation by mechanosensing GPCRs (105).

### Mechanosensing.

ECs can sense hemodynamic forces in a process termed mechanosensing, which is indispensable for vascular function. Disruptions in hemodynamics or in EC mechanosensing could significantly contribute to blood vessel dilation and/or CM lesion formation. Gαq/11-coupled GPCRs (GqPCRs) can participate in the mediation of mechanochemical signaling by (a) direct activation of PI3K/AKT, which can promote vasodilation via NO synthesis; (b) via PIP_2_, DAG, and IP_3_ metabolites that can modulate ion channels; and (c) by inducing membrane hyperpolarization.

Shear stress can activate bradykinin 2 (B2) receptors (106), leading to an increase in intracellular calcium levels that promote increased NO production or membrane hyperpolarization (107, 108). G protein–coupled receptor 68 (GPR68) and histamine H1 receptor (H1R) can instead mediate flow-induced vasodilation by enhancing NO production (109, 110).

GqPCR-initiated changes in intracellular metabolite levels can signal via EC-specific ion channels such as TRPV4, TREK-1, and Kir2.1 (111). Endothelial PIEZO1 is a mechanosensory ion channel activated by laminar flow. PIEZO1 can influence GqPCR activity and subsequent NO production by the release of ATP (105). Recent studies have implicated PIEZO1 in endothelial calcium signaling in the brain microvasculature (112). Therefore, dysregulation of PIEZO1 may be involved in CM vessel blood flow abnormalities and in vascular calcifications identified in SWS patients.

Studies on the effects of the *GNAQ* mutations in EC mechanosensors and mechanotransductors could reveal important associations and dysfunctions. Devising in vitro microfluidic systems with the use of *GNAQ* p.Q183R mutant ECs subjected to different types of flow dynamics and shear stress could advance our understanding of the regulation of the mechanosensing machinery in CM.

## Treatments, targets, and future therapies

Treatment for CM, particularly on the face, is focused on the prevention of progressive skin changes, including darkening and thickening that can result in disfigurement and even functional issues with age. Facial CMs are often treated with laser therapy, the most common of which is pulsed-dye laser. More recently, laser has been combined with therapeutic drugs such as sirolimus, delivered topically (113) or orally (114). The goal is to decrease rebound vasculogenesis after laser treatment and thereby increase efficacy of treatments. Treatment of SWS is also largely symptomatic, focused on seizure control with antiepileptics and on preventing thrombosis in the alternate draining vessels with antiplatelet treatment such as aspirin. Presymptomatic treatment with antiepileptics and/or aspirin is a recent focus among neurologists (115, 116), with hopes to delay or ameliorate seizure onset (11). This is of particular interest since later onset of seizure has been correlated with improved cognitive outcomes as compared with earlier-onset seizures (117, 118).

The identification of pathogenic variants in *GNAQ* in SWS and CM represents a potential new target for therapy. While the majority of therapies and trials for SWS to date have aimed at treating and controlling symptoms, usually seizures, identification of the underlying pathogenesis opens the door to development of new targeted therapies, or repurposing of existing drugs from oncology, as many of these same pathways are upregulated in human cancers.

One potential concern with any systemic therapy for SWS, with its most severe manifestations occurring in the brain, is the blood-brain barrier (BBB) and the ability to deliver medications to affected areas. However, one of the manifestations of vascular dysregulation in affected vascular beds is vessel instability and leakiness (VEGF-A–induced permeability and increased ANGPT2 expression). This has been described in epilepsy in general (119) and hypothesized in SWS. This localized breakdown of the BBB could be leveraged to deliver medications preferentially to the abnormal, affected vascular beds.

As discussed above, Gαq classically signals through PKC to activate the MAPK pathway and mild ERK activation was demonstrated in *GNAQ* p.R183Q mutant ECs and in patient samples (19, 73). It is well understood, however, that there is significant crosstalk between the RAS/RAF/MAPK and PI3K/AKT/mTOR pathways. While AKT was not shown to be upregulated in ECs expressing *GNAQ* p.R183Q (19), phospho-S6, a known effector of mTOR, was enriched in SWS brain tissue as compared with non-SWS epilepsy controls (120). A small pilot trial of 10 patients with SWS treated with sirolimus demonstrated safety in the SWS population (121). Furthermore, this study suggested that sirolimus might have a positive effect on patients with frequent stroke-like episodes, with decreased severity and duration of stroke-like symptoms, as well as neurocognitive changes, including improved processing speed and improvement in asymmetry on quantitative EEG.

MEK-directed therapies have now been used in a number of vascular anomalies, including for AVMs with *KRAS* mutations (122) or CM-AVM syndrome with *EPHB4* mutation (123), and in KLA with *NRAS* (124) and *CBL* (125) mutations. However, this has not been applied to patients with SWS to date.

Ideal future therapies would include agents that are more specifically targeted to upstream effectors of the dysregulated pathways. While direct Gαq inhibitors are still in preclinical development for uveal melanoma (126), PKC inhibitors are currently in phase I/II trials (reviewed in ref. 127).

In SWS patients, the frequency and intensity of the calcifications are likely to increase with age (128, 129), suggesting that early therapeutic intervention to prevent microvascular calcification could be efficacious in these patients. CRAC channel inhibitors are currently in early-phase clinical trials for inflammatory/autoimmune indications (130) and may be further investigated in the setting of SWS in the future.

As our understanding of the genetic origins and signaling consequences of CM continue to evolve, our therapeutic options will also continue to expand. New therapies may one day allow us to substantially improve the lives of people severely affected by SWS, as well as their families, and provide hope to halt or even reverse this often progressive process.

## Version 1 04/15/2024

Electronic publication
